# Involvement of the Cortico-Basal Ganglia-Thalamocortical Loop in Developmental Stuttering

**DOI:** 10.3389/fpsyg.2019.03088

**Published:** 2020-01-28

**Authors:** Soo-Eun Chang, Frank H. Guenther

**Affiliations:** ^1^Department of Psychiatry, University of Michigan, Ann Arbor, MI, United States; ^2^Department of Radiology, Cognitive Imaging Research Center, Michigan State University, East Lansing, MI, United States; ^3^Department of Communicative Sciences and Disorders, Michigan State University, East Lansing, MI, United States; ^4^Department of Speech, Language and Hearing Sciences, Sargent College of Health and Rehabilitation Sciences, Boston University, Boston, MA, United States; ^5^Department of Biomedical Engineering, Boston University, Boston, MA, United States; ^6^Picower Institute for Learning and Memory, Massachusetts Institute of Technology, Cambridge, MA, United States; ^7^Department of Radiology, Massachusetts General Hospital, Charlestown, MA, United States

**Keywords:** stuttering, basal ganglia thalamocortical circuitry, pathophysiology, theoretical modeling coupled with experimental approachest, magnetic resonance imaging

## Abstract

Stuttering is a complex neurodevelopmental disorder that has to date eluded a clear explication of its pathophysiological bases. In this review, we utilize the Directions Into Velocities of Articulators (DIVA) neurocomputational modeling framework to mechanistically interpret relevant findings from the behavioral and neurological literatures on stuttering. Within this theoretical framework, we propose that the primary impairment underlying stuttering behavior is malfunction in the cortico-basal ganglia-thalamocortical (hereafter, cortico-BG) loop that is responsible for initiating speech motor programs. This theoretical perspective predicts three possible loci of impaired neural processing within the cortico-BG loop that could lead to stuttering behaviors: impairment within the basal ganglia proper; impairment of axonal projections between cerebral cortex, basal ganglia, and thalamus; and impairment in cortical processing. These theoretical perspectives are presented in detail, followed by a review of empirical data that make reference to these three possibilities. We also highlight any differences that are present in the literature based on examining adults versus children, which give important insights into potential core deficits associated with stuttering versus compensatory changes that occur in the brain as a result of having stuttered for many years in the case of adults who stutter. We conclude with outstanding questions in the field and promising areas for future studies that have the potential to further advance mechanistic understanding of neural deficits underlying persistent developmental stuttering.

## Introduction

Developmental stuttering (for brevity, “stuttering” hereafter) is a childhood onset speech disorder that affects approximately 5–8% of children and 1% of adults (Månsson, 2000; Reilly et al., 2009). Core symptoms of stuttering include involuntary, frequent disruptions during ongoing speech such as part-word repetitions, sound prolongations, and silent blocks, which interrupt fluent speech and impair communication (Bloodstein and Ratner, 2008). While considered a disorder affecting speech motor control, stuttering is a distinct disorder from dysarthrias, in that there is no underlying weakness or paralysis of the articulatory musculature, and apraxia of speech (AOS), in that stuttering involves the interruptions of flow described above with otherwise intact productions of intended sounds, whereas AOS involves uncoordinated movements, distorted productions, omissions, and substitutions of sounds.

Stuttering can be either *neurogenic*, arising through stroke, neurological disease, or as a result of treatments for neurological diseases (see Lundgren et al., 2010; Theys et al., 2013; Craig-McQuaide et al., 2014 for reviews), or, more commonly, *developmental*, typically emerging at 2–5 years of age in an estimated 3–8% of preschool-aged children but resolving spontaneously within 2 years in 75% of cases (Yairi et al., 1996; Curlee, 2004; Yaruss and Quesal, 2004; Yairi and Ambrose, 2013). Those cases that do not resolve are referred to as *persistent developmental stuttering* (PDS), which affects approximately 1% of the population and occurs in nearly all cultures and languages (Van Riper, 1982).

As reviewed in later sections, anomalies in a bewildering array of neural structures have been identified in people who stutter. Craig-McQuaide et al. (2014) provided an earlier comprehensive review of basal ganglia in the context of its possible role in pathophysiology of both developmental and neurogenic stuttering. In the current review, we will utilize the Directions Into Velocities of Articulators (DIVA) neurocomputational modeling framework (Guenther et al., 2006; Bohland et al., 2010; Guenther, 2016) to mechanistically interpret relevant findings from the behavioral and neurological literatures on developmental stuttering. The DIVA model divides speech into feedforward and sensory feedback-based control processes. The feedforward control system is further sub-divided into an *articulation circuit*, which is responsible for generating the finely timed and coordinated muscle activation patterns (*motor programs*) for producing speech sounds, and an *initiation circuit*, which is responsible for turning the appropriate motor programs on and off at the appropriate instants in time. Following seminal work from the primate motor control literature (Alexander et al., 1986; Mink, 1996), the initiation circuit is hypothesized to involve the cortico-basal ganglia-thalamocortical loop (hereafter referred to as the *cortico-BG loop*). Within our theoretical framework, then, *the primary impairment underlying stuttering behavior is malfunction in the cortico-BG loop* responsible for initiating speech motor programs (see also Alm, 2004; Craig-McQuaide et al., 2014). This theoretical perspective is further detailed in the next section, followed by a review of related empirical findings regarding neural processing in individuals who stutter.

## The Cortico-Basal Ganglia-Thalamocortical Loop and Stuttering: Theoretical Perspectives

**Figure 1:**
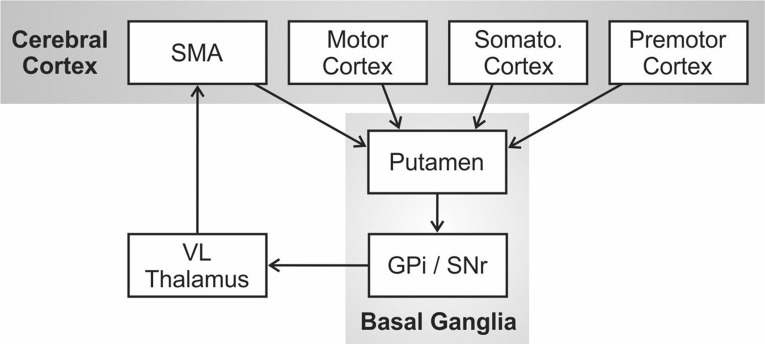
The cortico-basal ganglia motor circuit as originally proposed by Alexander et al. (1986). Abbreviations: GPi = internal segment of the globus pallidus; SMA = supplementary motor area; Somato = somatosensory; SNr = substantia nigra pars reticulata; VL = ventral lateral nucleus.

Alm (2004) proposed that the core deficit in PDS is an impaired ability to initiate, sustain, and/or terminate motor programs for phonemic/gestural units within a speech sequence due to impairment of the left hemisphere cortico-BG loop^1^. The cortico-BG loop was originally described as one of several distinct functional circuits in the primate brain involving loops from cerebral cortex to the basal ganglia, thalamus, and back to cortex by Alexander et al. (1986). This circuit is schematized in Figure 1. Mink (1996) further hypothesized that the role of this circuit was not to directly generate movements but instead to select (or dis-inhibit) the correct movement under the current behavioral circumstances while inhibiting the competing movements.

Neural pathways for speech and vocal learning may have evolved adjacent to, or embedded in, motor learning pathways that are commonly present in both vocal (e.g., humans, songbirds) and non-vocal learning (e.g., non-human primates) species (Feenders et al., 2008; Jarvis, 2019). Vocal learning pathways and song nuclei in songbirds – although cell types and cortical organization differ – parallel brain areas within the cortico-BG motor circuitry involved in speech production in humans. Specifically, the anterior vocal pathway (also referred to as the anterior forebrain pathway or the anterior song pathway) of songbirds encompasses a cortical-striatal-thalamic loop that connects a human premotor cortex homologue (LMAN) to the striatum (Area X) and ventral lateral nucleus of the thalamus in the songbird brain (Chakraborty et al., 2017; Jarvis, 2019). The anterior vocal pathway projects directly to the posterior vocal motor pathway (comprising the human Broca’s and ventral motor cortex homologues) and contributes to improving motor pathway performance by generating error correction signals that reduce vocal error (Andalman and Fee, 2009; Gadagkar et al., 2016; Kojima et al., 2018). The anterior vocal pathway and the direct connections between motor cortex and brainstem vocal motor neurons that enable fine motor control of vocalization are characteristic of vocal learning species that are able to imitate and modify sounds and undergo a period of sensorimotor song learning from tutors (as opposed to “vocal non-learning species” such as non-human primates that produce only innate vocalizations). Disrupting function in critical parts of this pathway, for instance, by lesioning the striatal homologue area X (Kubikova et al., 2014) or over-expression of a gene affecting LMAN function (Chakraborty et al., 2017), induced stuttering in songbirds. The songbirds exhibited increased repetition of syllables at the end of song motifs, usually the first motif of a bout, indicating that the birds were being stuck in transitioning from one motif sequence to the next. This behavior is comparable to what is seen in human stuttering, where timing, initiation, and sequencing of syllables are posited to be affected *via* aberrant BG-cortical function (Alm, 2004).

In the DIVA model, the initiation circuit is responsible for sequentially initiating phonemic gestures within a (typically syllabic) motor program by activating nodes for each phoneme in an initiation map in the supplementary motor area (SMA), a region of cerebral cortex thought to be involved in the initiation of motor programs, including those for speech (Jonas, 1987; Ziegler et al., 1997). In terms of information flow in Figure 1, the premotor, motor, and somatosensory cortical regions involved in movement planning and execution are, in effect, being monitored by the basal ganglia *via* projections from cortex to the putamen (the primary input nucleus of the basal ganglia for motor processing). Internal circuitry within the basal ganglia, including portions of the globus pallidus (GP) and substantia nigra (SN), performs the job of selectively exciting the correct motor program in the current context while inhibiting the competing motor programs. For example, if a mature, fluent speaker is currently producing the word “pet” and is in the process of producing “p,” the basal ganglia are monitoring the sensorimotor representations of the speech articulators for evidence of the impending completion of “p” (e.g., lip contact); when this occurs, a “completion signal” is sent to cerebral cortex to extinguish the initiation map node for “p”, at which time the cortico-BG loop selects “e” over competing motor programs and sends this information to SMA to activate the initiation map node for “e.”

**Figure 2:**
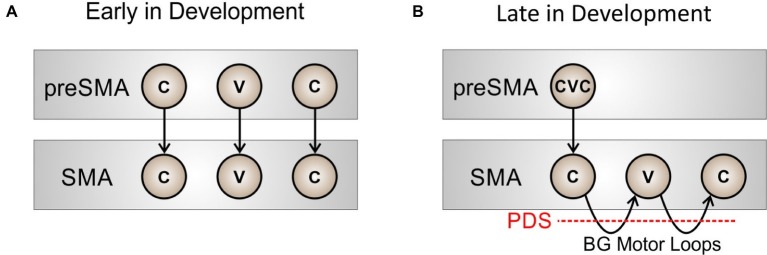
Schematized view of the process of sequencing through phonemes in the word “pet” at two developmental stages: **(A)** early in development, when pre-SMA involvement is required to sequentially activate nodes in SMA for initiating each phoneme, and **(B)** later in development, when the basal ganglia motor loop has taken over sequential activation of the SMA nodes.

Before a new sequence (syllable) is fully learned by the basal ganglia, the motor system will rely heavily on cortical mechanisms to sequence through phonemic motor programs. This situation is schematized in Figure 2A for the word “pet.” Very early in development (prior to the age of 2–3 years), initiation of the phonemes in “pet” requires relatively high-level cortical input from pre-SMA (a region known to be involved in motor sequencing; e.g., Shima and Tanji, 2000) to sequentially activate the proper initiation map nodes. With repeated practice, the basal ganglia motor loop will take over the load of sequencing through the individual phonemes in the word, as in Figure 2B, thus making production more “automatic” and freeing up higher-level cortical areas such as pre-SMA. Within this view, stuttering can be interpreted as an impairment of the cortico-BG loop’s role in initiation and sequencing of learned speech sequences, as indicated by the red dashed line in Figure 2B.

**Figure 3:**
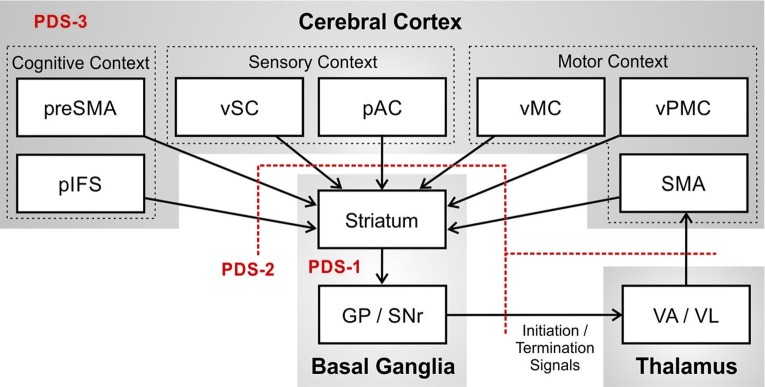
Potential impairments of the basal ganglia motor loop that may contribute to persistent developmental stuttering (PDS), specifically the basal ganglia (PDS-1); axonal projections between cerebral cortex, basal ganglia, and thalamus (PDS-2); and the network of cortical regions involved in speech (PDS-3). (Abbreviations: GP = globus pallidus; pAC = posterior auditory cortex; pIFS = posterior inferior frontal sulcus; pre-SMA = pre-supplementary motor area; SMA = supplementary motor area; SNr = substantia nigra pars reticulata; VA = ventral anterior thalamic nucleus; VL = ventral lateral thalamic nucleus; vMC = ventral motor cortex; vPMC = ventral premotor cortex; vSC = ventral somatosensory cortex).

Figure 3 provides an expanded view of the basal ganglia motor loop. The basal ganglia in essence performs a pattern matching operation in which it monitors the current cognitive context as represented by activity in prefrontal cortical areas including pre-SMA and the posterior inferior frontal sulcus (pIFS); motor context represented in ventral premotor cortex (vPMC), SMA, and ventral primary motor cortex (vMC); and sensory context represented in posterior auditory cortex (pAC) and ventral somatosensory cortex (vSC). When the proper context is detected, the basal ganglia signals to SMA that means it is time to terminate the ongoing phoneme (termination signal) and initiate the next phoneme of the speech sequence (initiation signal).

Failure to recognize the sensory, motor, and cognitive context for terminating the current phoneme would result in a prolongation stutter since activity of the SMA initiation map node for the current sound will not be terminated at the right time. Failure to recognize the context for initiating the next phoneme would result in a block stutter since the initiation map node for the next phoneme in SMA will not be activated at the right time. If the initiation signal “drops out” momentarily, production of the next phoneme might begin but prematurely terminate and then restart, as in a repetition stutter. Figure 3 also indicates, in red, three distinct (but not mutually exclusive) loci of impaired neural processing that could lead to these stuttering behaviors: impairment within the basal ganglia proper (PDS-1); impairment of axonal projections between cortex, basal ganglia, and thalamus (PDS-2); and impairment in cortical processing (PDS-3). The review of empirical data in the next section will make reference to these three possibilities.

Alm (2004) refers to signals such as the initiation and termination signals discussed above as *timing signals*, since they indicate the right time to terminate/initiate movements. This characterization provides an insight into the frequent observation that stuttering is often greatly reduced or eliminated in situations where external timing cues are available, such as choral reading and metronome-timed speech (Bloodstein, 1995). Interpreted within the DIVA/GODIVA framework, these tasks involve timing signals that are perceived by sensory cortical areas, which then relay the signals to SMA, thereby reducing dependence on the basal ganglia motor loop for generating initiation/termination signals. Singing, which also increases fluency in PDS (Starkweather, 1987), likely involves different mechanisms for generating phonemic timing than the basal ganglia motor loop used for initiating propositional speech.

## The Cortico-Basal Ganglia-Thalamocortical Loop and Stuttering: Empirical Findings

### Impairment in the Basal Ganglia Proper (PDS-1)

The basal ganglia are frequently associated with stuttering in the speech production literature. For example, neurogenic stuttering is often associated with damage to the left caudate nucleus and putamen (Theys et al., 2013). Furthermore, stuttering often develops or re-emerges in Parkinson’s disease (Koller, 1983; Leder, 1996; Benke et al., 2000; Shahed and Jankovic, 2001; Lim et al., 2005), the motor components of which are thought to arise from impairment of function within basal ganglia structures as described earlier. Deep brain stimulation applied to the STN of the basal ganglia can relieve acquired stuttering in some Parkinson’s disease patients (Walker et al., 2009; Thiriez et al., 2013), while in others, it seems to exacerbate stuttering (Burghaus et al., 2006; Toft and Dietrichs, 2011). Levodopa treatment, aimed at increasing dopamine levels in the striatum of the basal ganglia, can also exacerbate stuttering (Anderson et al., 1999; Louis et al., 2001; Tykalová et al., 2015).

This last finding fits well with one popular hypothesis regarding the neurogenesis of PDS, the *dopamine excess theory*, which is based on the Wu et al. (1997) finding of excessive dopamine in the striatum of three PWS compared to six non-stuttering control participants^2^. Computer simulations performed by Civier et al. (2013) verified that an increased level of dopamine in the striatum can lead to stuttering behaviors in a version of the DIVA model that includes higher-level speech sequencing circuitry, called the GODIVA model. To understand how this can occur, it is useful to note that there are two largely distinct pathways within the basal ganglia: a *direct pathway* that has the overall effect of exciting cerebral cortex (needed to activate the correct motor program) and an *indirect pathway* that has the overall effect of inhibiting cerebral cortex (needed to suppress competing motor programs). Striatal dopamine has opposite effects on the two pathways: it excites the direct pathway and inhibits the indirect pathway. Thus, excessive dopamine can lead to a situation in which there is insufficient inhibition to suppress competing motor programs, making it difficult for the correct motor program to be chosen over incorrect alternatives. Such a situation could delay the choice of the desired motor program, leading to a block or prolongation stutter, or it may lead to an unstable initiation signal that starts to increase but suffers dropouts that result in repetition stutters.

In support of the dopamine excess theory of stuttering, it has been noted that antipsychotic drugs such as haloperidol and risperidone that block dopamine D2 striatal receptors are effective in treating symptoms of stuttering^3^ (see Bothe et al., 2006, for a review). These D2 antagonists increase the efficacy of the indirect pathway by removing it from dopaminergic inhibition, thus correcting the hypothesized direct/indirect imbalance and increasing the inhibition of competing actions. A weakened indirect pathway and concomitant inability to maintain the chosen action over competing actions are also supported by the study of Webster (1989) demonstrating that PWS are particularly impaired in initiating and progressing through sequences in the presence of competing tasks. Alm (2004) suggests that developmental changes in dopamine receptor density in the putamen could also explain the pattern of early childhood onset and recovery, including gender differences.

Relatedly, the computer simulations of Civier et al. (2013) also indicate that *decreased* dopamine levels in the striatum could lead to stuttering dysfluencies, which would account for the onset of stuttering in individuals with Parkinson’s disease noted above. In this scenario, reduced excitation of the desired motor program through the direct pathway leads to a decrease in the competitive advantage of this motor program, which in turn leads to a delayed, weakened, and/or unstable initiation signal. It should be noted that many people with PD exhibit speech disruptions other than stuttering, further highlighting that the relationship between Parkinson’s disease and stuttering is not a simple one. Studies aimed at distinguishing the neural characteristics of PD patients with stuttering-like behaviors from those with other types of speech motor disruptions may help clarify this relationship.

These considerations suggest that there may be at least two subtypes of PDS: one characterized by an under-active indirect pathway and another characterized by an under-active direct pathway. Behaviorally, the former might be characterized by a tendency toward excessive motor activity due to reduced inhibition of movement from the indirect pathway, whereas the latter might be characterized by a reduced level of motor activity due to reduced excitation of movement from the direct pathway. This is similar to the proposal put forth by Alm (2004), who proposed a breakdown into D2-responsive and stimulant-responsive subgroups of PWS. It should be noted, however, that our treatment of basal ganglia anatomy and physiology has been highly schematic, and that the actual situation is very complex, involving many neurotransmitter types and axonal pathways in addition to those discussed herein. Nonetheless, there is sufficient evidence for the differentiation of stuttering subtypes involving different malfunctions of the basal ganglia to merit increased research on this topic, including large-sample studies investigating striatal dopamine levels in PDS.

Further evidence of possibly impaired basal ganglia functioning in PDS comes from a functional magnetic resonance imaging (fMRI) study by Giraud et al. (2008), who found that neural activity during speech in the striatum (specifically the head of the caudate nucleus, which lies immediately anterior to the putamen) was positively correlated with stuttering severity in 16 adults with PDS, and that this correlation largely disappeared after 3 weeks of intensive therapy. Possible impairment of the striatum (in this case the putamen) was also identified using voxel-based morphometry by Lu et al. (2010), who found increased gray matter volume concentration in the left putamen of adults who stutter compared to controls.

### Impairments in Projections Between Cerebral Cortex, the Basal Ganglia, and Thalamus (PDS-2)

The second potential source of impairment in the basal ganglia motor loop of PDS identified in Figure 3, labeled PDS-2, is the set of projections from cerebral cortex to striatum that convey the current sensorimotor and cognitive context to the basal ganglia. Computer simulations of the GODIVA model by Civier et al. (2010, 2013) indicate that impaired corticostriatal connectivity can result in poor detection of the cognitive and sensorimotor context for initiating the next sound by the basal ganglia motor loop, thereby impairing the generation of initiation/termination signals to SMA. It is thus tempting to conclude that impaired left hemisphere corticostriatal connectivity may be a root cause of stuttering.

Neuroimaging results from several studies provide some support for this contention. Using diffusion tensor imaging (DTI) data acquired from CWS and age-matched controls, Chang and Zhu (2013) found that CWS have less structural connectivity between left putamen and several left hemisphere cortical regions, including IFo and SMA. In another DTI study, Chow and Chang (2017) reported decreased growth rate in a measure reflecting white matter integrity (fractional anisotropy; FA) in children with PDS in the anterior thalamic radiation, which connects the prefrontal areas with the cortico-BG loop (Alexander and Crutcher, 1990; Hélie et al., 2015). Although the role of this circuit in speech production is not fully understood, it has been implicated in sequence learning (Graybiel, 2005), rule-based categorization (Ell et al., 2010; Ashby et al., 2011), attention switching (Ravizza and Ciranni, 2002), and working memory (Taylor and Taylor, 2000; Voytek and Knight, 2010). The anomalies in the connections between prefrontal areas and the basal ganglia may affect higher-order cognitive functions (e.g., attention), which help establish and later develop speech control automaticity *via* the cortico-BG loop. This interpretation is also relevant to the Chow and Chang (2017) findings that show a negative relationship between stuttering severity and FA along the anterior and superior thalamic radiations in PDS. Specifically, lower FA in these tracts was associated with more severe stuttering in children with PDS. These results suggest that attenuated FA in tracts interconnecting frontal areas and the cortico-BG loop, which helps interface speech motor control and other cognitive functions, may contribute to severity and persistence in stuttering.

Atypical processing in corticostriatal circuits has also been shown through functional connectivity analyses of resting state fMRI data. In one study (Chang et al., 2016), the relationship between rhythm perception and timing-related brain network activity was examined. Rhythm processing is a skill that underlies not only rhythm perception but also speech perception and production (Kotz and Schwartze, 2010). In fluent children, correlated activity patterns involving the putamen and cortical areas within the cortico-BG loop (including premotor, motor, SMA, and auditory cortex) were associated with performance of a rhythm discrimination task, which requires proficient processing of timing information of auditory events. Namely, the extent of functional connectivity among these brain areas was strongly correlated with performance on the rhythm discrimination task. In the case of CWS, the strong association between functional connectivity and rhythm discrimination performance observed in controls was absent. This finding is suggestive of a deficit in the ability to perceive temporally structured sound sequences in CWS.

Compared to CWS, clear evidence of impaired cortico-subcortical connectivity in adults who stutter remains relatively scarce. However, Lu et al. (2010) used structural equation modeling (SEM) of brain activity during an fMRI picture naming task to identify anomalous functional connectivity in several pathways of the cortico-BG loop in adults who stutter, including pathways between auditory cortical areas and putamen and thalamus, between thalamus and pre-SMA, and between thalamus and putamen.

### Impairments in the Network of Cortical Regions That Process Cognitive and Sensorimotor Aspects of Speech (PDS-3)

The third possible source of impairment in the basal ganglia motor loop of PDS is the network of cerebral cortical regions involved in speech production (PDS-3 in Figure 2). Neurogenic stuttering is generally associated with damage to speech-related areas in the left (language-dominant) cortical hemisphere in addition to the left striatum (Theys et al., 2013). Likewise, structural differences in the left inferior frontal and premotor cortex regions have been repeatedly reported for developmental stuttering, both in children and in adults (e.g., Chang et al., 2011; Beal et al., 2012). This suggests that stuttering involves prefrontal and/or premotor cortical mechanisms for speech, which, unlike primary sensory and motor cortical areas that show relatively little hemispheric differentiation, are predominantly located in the left hemisphere.

Structural neuroimaging studies of PDS further support this assertion. For example, Kronfeld-Duenias et al. (2016) used diffusion tensor imaging (DTI) to identify anomalous diffusivity of white matter in the left frontal aslant tract (FAT) of adults who stutter that correlated with stuttering severity; this tract connects medial premotor areas such as SMA and pre-SMA with posterior inferior frontal cortical areas (Dick et al., 2013) that are associated with speech motor programs in the DIVA model. Relatedly, Kemerdere et al. (2016) found that axonal stimulation of the FAT, which transiently “lesions” the tract, led to transitory stuttering.

**Figure 4:**
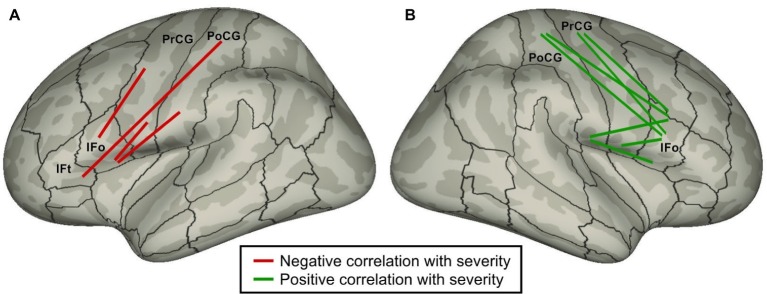
Schematic of intra-hemispheric white matter tracts between inferior frontal cortical regions and Rolandic cortical regions whose strengths are significantly correlated with stuttering severity (Cai et al., 2014) plotted on **(A)** left and **(B)** right lateral inflated cortical surfaces. Red tracts indicate a negative correlation with severity (i.e., weaker tracts are associated with higher severity); green tracts indicate a positive correlation. (Abbreviations: IFo = inferior frontal gyrus pars opercularis; IFt = inferior frontal gyrus pars triangularis; PoCG = postcentral gyrus; PrCG = precentral gyrus).

Cai et al. (2014) used DTI and probabilistic tractography to identify correlations between stuttering severity and white matter tract strengths in PDS. It is commonly believed that, all else equal, stronger white matter tracts are associated with better performance, and that white matter structural changes correlate with learning/training (Scholz et al., 2009; Zatorre et al., 2012). According to this view, if a particular tract is part of the underlying cause of stuttering, we would expect that the weaker the tract, the more severe the stuttering, i.e., tract strength should be negatively correlated with stuttering severity. Conversely, the strength of a tract that is forced into action to (incompletely) compensate for the core neural impairment should be positively correlated with severity. Figure 4 indicates all intra-hemispheric tracts between inferior frontal cortical ROIs and sensorimotor (Rolandic) cortical ROIs that were significantly correlated with severity in the work of Cai et al. (2014). Strikingly, all such tracts in the left hemisphere were negatively correlated with stuttering, while all right hemisphere tracts were positively correlated. This finding suggests that impaired performance in the left hemisphere cortical network for speech in PDS forces reliance on right hemisphere homologues, leading to increased right hemisphere white matter tract strengths due to additional use. This interpretation is a variant of the atypical cerebral laterality view of stuttering that dates as far back as Orton (1927). Interpreted within the DIVA/GODIVA framework, the left hemisphere white matter impairments are indicative of impaired function of the left-lateralized feedforward control system, resulting in sensory errors that must be corrected by the right-lateralized auditory and somatosensory feedback control systems.

Further support for the view that left hemisphere impairments in PDS result in increased right hemisphere involvement during speech comes from functional neuroimaging studies. Anomalous functioning in left hemisphere inferior frontal cortex in adults with PDS during single-word production was identified using magnetoencephalography by Salmelin et al. (2000), who also noted that suppression of motor rhythms (which reflect task-related processing) was right dominant in PDS but left dominant in fluent speakers. Hyperactivity in right hemisphere cerebral cortex of PWS has been noted in a number of prior PET and fMRI studies (e.g., Fox et al., 1996; Braun et al., 1997; Ingham et al., 2000; De Nil et al., 2001; Neef et al., 2018). The view that right hemisphere cortical hyperactivity results from impaired left hemisphere function is also consistent with the effects of fluency-inducing therapy on BOLD responses; successful treatment has been associated with a shift toward more normal, left-lateralized frontal activation (De Nil et al., 2003; Neumann et al., 2005).

**Figure 5:**
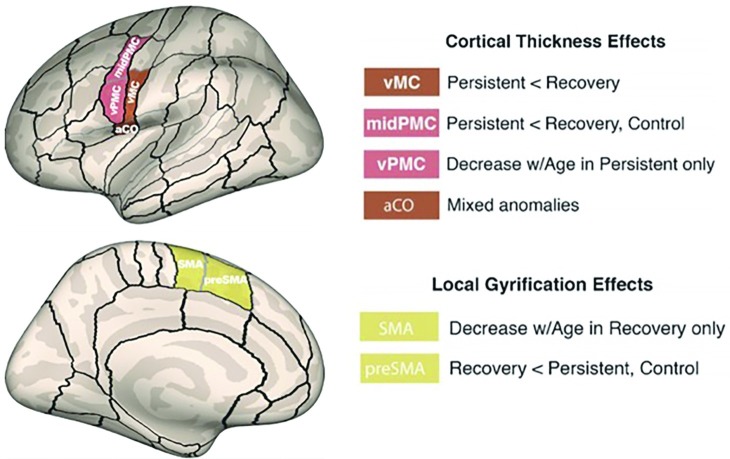
Morphometric differences in speech motor control regions differentiated children with persistent stuttering from those who recover. A compensatory mechanism involving left medial premotor cortex may contribute to recovery (Garnett et al., 2018). Reprinted from Garnett et al. (2018), by permission of Oxford University Press. Copyright © 2018 Oxford University Press.

Consistent with the view that left hemisphere anomalies underlie PDS, Garnett et al. (2018) identified several left hemisphere differences in cortical morphology of CWS compared to age-matched controls. Surface-based measures of cortical thickness and gyral anatomy were extracted in perisylvian and dorsal medial regions relevant to speech processing (Tourville and Guenther, 2003). The results showed that children with persistent stuttering had significantly decreased cortical thickness in left ventral motor cortex (vMC) and ventral premotor cortex (vPMC) areas relative to controls (Figure 5). vMC contains representations of the speech articulators, including the larynx (in particular, the ventral laryngeal representation; cf. Belyk and Brown, 2017), tongue, jaw, and lips (see Guenther, 2016, Appendix A for a review). This decreased thickness was not found in children who eventually recovered from stuttering. Recovered children had decreased gyrification^4^ in the SMA and pre-SMA areas with increasing age, which may indicate better long-range connectivity with regions such as left IFG.

In the first longitudinal DTI study in childhood stuttering, Chow and Chang (2017) showed that white matter integrity in major tracts such as the left arcuate fasciculus was decreased in CWS relative to their fluent peers. Specifically, sections along the left arcuate fasciculus underlying the temporoparietal junction and posterior temporal areas were decreased in CWS regardless of their eventual persistence or recovery. Furthermore, significant age-related white matter integrity increases were found in these same areas in the recovered group, but this was not the case for the persistent group. Namely, growth trajectories normalized with age in the recovered group but stagnated in the persistent group. This suggests that normalized structural connectivity among left premotor, motor, and auditory cortical areas may play a role in natural recovery from stuttering in childhood. The commonly reported finding of decreased FA affecting the frontal motor areas in stuttering speakers relative to fluent speakers (Sommer et al., 2002; Chang et al., 2008, 2011; Watkins et al., 2008; Cykowski et al., 2010) was also reported in this study.

Another common finding in adults with PDS is reduced neural activity in left hemisphere auditory cortex of the posterior superior temporal gyrus compared to fluent controls (e.g., Fox et al., 1996, 2000; Braun et al., 1997; De Nil et al., 2001). Auditory cortical activity impacts motor actions *via* corticostriatal projections (Znamenskiy and Zador, 2013). Thus, if auditory feedback of one’s own speech does not match the expected pattern for the current sound (due, for example, to subtle errors in articulation), the striatum may detect a mismatch between the current sensorimotor context and the context needed for initiating the next motor program, thus reducing its competitive advantage over competing motor programs, which in turn may lead to impaired generation of initiation signals by the basal ganglia and a concomitant stutter.

This view receives support from a number of findings. First, it has long been known that there is a very low rate of stuttering in congenitally deaf individuals (e.g., Backus, 1938; Harms and Malone, 1939; Van Riper, 1982). Furthermore, a number of manipulations that interfere with normal auditory feedback processing of one’s own speech can alleviate stuttering, including noise masking (Maraist and Hutton, 1957; Adams and Hutchinson, 1974), chorus reading (Barber, 1939; Kalinowski and Saltuklaroglu, 2003), pitch-shifted auditory feedback (Macleod et al., 1995), and delayed auditory feedback (Stephen and Haggard, 1980). These conditions may have the effect of eliminating the detection of small errors in articulation that would otherwise reduce the match between expected and actual sensorimotor context for the next motor program in striatum. In light of these considerations, the reduced activity in auditory cortex of adults who stutter may reflect a compensatory mechanism involving inhibition of auditory feedback of one’s own speech to avoid detection of minor errors in production. This conjecture receives some support from findings of reduced responses to auditory perturbations during speech in adult PWS compared to age-matched controls (e.g., Cai et al., 2012, 2014; Daliri et al., 2018). Interestingly, Daliri et al. (2018) found that CWS did not show a reduction in adaptation to auditory perturbation compared to non-stuttering children, suggesting that increased inhibition of auditory feedback during speech may develop gradually in PWS as a means to reduce dysfluency.

To date this conjecture has received relatively little support from neural studies of CWS, primarily due to difficulties in performing task-related functional neuroimaging in young children near the age of onset of stuttering. However, Walsh et al. (2017) used functional near infrared spectroscopy (fNIRS) to measure hemodynamic responses in CWS and age-matched controls performing a picture description task that induced continuous speech production. While the fluent controls showed the expected neural activity in the left inferior frontal and premotor cortices during this task, a deactivation in the same areas was observed in the case of CWS. There were no significant group differences found in the auditory areas, providing some support for the idea that deactivation of auditory cortex is a compensatory mechanism developed after years of stuttering rather than a root cause of the disorder.

## Discussion

In this paper, we reviewed theoretical perspectives and extant empirical data substantiating the possible critical role of the cortico-BG loop in stuttering etiology. Impairment of this loop was posited to take three different possible forms: deficits in the basal ganglia proper, deficits in the connections between the main neural structures of the cortico-BG loop (cortex, basal ganglia, and thalamus), and deficits in the cerebral cortex. Neuroimaging data to date from both children and adults who stutter have provided evidence to support deficits in each of these areas relative to fluent speakers. The differences that are present in the literature based on examining adults versus children give important insights into potential core deficits associated with stuttering versus compensatory changes that occur in the brain as a result of having stuttered for many years in the case of adults who stutter. Below we discuss some promising areas of investigation that have the potential to further our understanding of the role of the basal ganglia in stuttering pathophysiology.

### Differences Between Adults and Children Who Stutter Provide Important Insights Into Distinguishing Primary Deficits From Secondary Effects in Stuttering

Anatomical and functional anomalies involving the left hemisphere premotor cortex, IFG, SMA, and putamen have been found in both adults and children who stutter, suggesting that these impairments may represent the primary deficits underlying stuttering. By contrast, differences between adults and children who stutter have been identified, including auditory cortex deactivation relative to controls during speech and decreased compensation to auditory perturbations in adults who stutter but not CWS. Currently, this assertion has only been supported in a small number of studies involving CWS, but if this pattern holds up in additional studies, it suggests that decreased auditory feedback processing during speech in adults who stutter is a secondary effect that may develop over years of stuttering; perhaps, as a compensatory mechanism that decreases the probability that a stutter will be induced by the detection of minor inaccuracies in speech output through auditory feedback. More generally, studies that directly compare adults and CWS as well as longitudinal studies of individuals who stutter (including both persistent cases and those that resolve over time) are needed to tease apart primary deficits from the many secondary behaviors and neural anomalies that have been identified in adults who stutter. In turn, this knowledge will aid in the development of effective treatments aimed squarely at the root causes of the disorder.

### Network-Level Connectivity Analyses of the Cortico-Basal Ganglia-Thalamocortical Loop May Be Critical for Accurately Identifying and Characterizing Deficits in This Loop in Stuttering

The preceding review makes clear that neural anomalies in stuttering have been identified in a number of different portions of the cortico-BG loop. Furthermore, individual connectivity studies often report disparate neural pathways that differ in individuals who stutter compared to controls. These considerations are indicative of the fact that stuttering is likely a *system-level problem* rather than the result of impairment in a particular neural region or pathway.

One implication of this is that network-level analyses, such as those studied in graph theory, may provide a more reliable and effective means of identifying the neural bases of the disorder. For example, Cai et al. (2014) used network-based statistics to characterize connectivity anomalies in adults who stutter. This study identified a relatively large deficit in betweenness centrality of left vPMC within the speech network of adults who stutter, indicating that this region (which is normally the most “central” component of the speech network) plays a substantially less central role in the speech network of individuals who stutter, though the specific brain areas with which this area has decreased connectivity differ across individuals. In another study, Chang et al. (2018) used a whole-brain independent component analysis (ICA) of resting state fMRI data to show that CWS could be differentiated from fluent peers through examining how large-scale intrinsically connected networks interact within and between different canonical networks identified in prior resting state functional connectivity studies of neurotypical individuals (Damoiseaux et al., 2006; Spreng et al., 2013). This analysis was limited to cortical areas and was able to show that certain connectivity patterns during early years could predict later persistent stuttering in CWS. CWS in general showed aberrant connectivity patterns involving the somatomotor network and its connectivity with frontoparietal and attention networks. These findings have important implications for how attention could mediate corticocortical and corticostriatal connectivities that were discussed in earlier sections. The persistent CWS (but not the recovered CWS) also showed aberrant connectivity involving the default mode network (DMN) and its connections to attention and frontoparietal networks. These results suggest that cognitive and higher-order functions could be involved in mediating recovery or persistence in stuttering symptoms. The aberrant connectivity involving DMN indicates an immature pattern of network interaction that does not efficiently segregate between task positive (e.g., attention, frontoparietal, and somatomotor) and task negative (e.g., DMN) networks. It has been proposed in a “default network interference model” (Sonuga-Barke and Castellanos, 2007), that DMN intrudes on task-positive networks and adds variability in performance of externally directed tasks. Better segregation from task-negative networks to enable efficient functioning of the somatomotor, executive control, and attention networks could allow once-vulnerable children to recover from stuttering. Those who are not able to achieve normalized segregation among networks could have difficulty compensating for possibly aberrant cues from the basal ganglia by engaging auditory and motor areas. Future studies will look more closely into specific connectivity affecting the cortico-BG loop and better understand how speech motor control (cortical and subcortical areas) is affected by these large-scale networks.

### Examining Neural Oscillations Could Help Reveal the Nature of Neural Communication Deficits Observed in Stuttering

Apart from MRI-based studies, we expect that increasing attention will be given to the temporal dynamics of neural communication, including anomalies in these dynamics in stuttering speakers. According to the “communication through coherence” hypothesis of neural communication (Fries, 2005), neural oscillatory synchrony mediates communication between different neural structures and subsystems. Neural oscillations are categorized based on the characteristic frequencies at which the rhythms occur; among these, beta oscillations that occur in the 13–30 Hz range are prevalent in the motor system (Pogosyan et al., 2009; Joundi et al., 2012; Kilavik et al., 2013). Beta activity seems to cue the initiation and termination of a movement sequence, enabling internally driven timing of movement sequences (Bartolo and Merchant, 2015). Coherence in beta oscillations reflects functional coordination between auditory and motor systems and “dynamically configures the sensorimotor networks for auditory-motor coupling” (Fujioka et al., 2012, p. 1791). Furthermore, basal ganglia (striatal) beta activity reflects the utilization of sensory cues to guide behavior (Leventhal et al., 2012), indicating that beta activity might serve as a channel for the basal ganglia to modulate cortical auditory-motor interaction relevant to motor control.

Specific to speech, beta oscillations in the motor cortex during speech preparation reflect the communication of the speech plan to the motor effectors and to the sensory regions required for monitoring speech output (Bowers et al., 2013, 2018; Liljeström et al., 2015). Coherence in the beta range is observed between bilateral primary motor and premotor cortices and auditory cortex during speech preparation (Liljeström et al., 2015). Results suggesting aberrant neural oscillations involving beta and other frequency bands have been reported in both children (Özge et al., 2004; Etchell et al., 2016) and adults who stutter (Joos et al., 2014; Mersov et al., 2016; Mock et al., 2016; Sengupta et al., 2016; Kikuchi et al., 2017; Saltuklaroglu et al., 2017). In adults who stutter, beta desynchronization and synchronization, which occur characteristically during movement preparation and execution respectively, were both exaggerated relative to controls (Mersov et al., 2016). These results point to an abnormal neural coordination during speech preparation and execution in stuttering.

In summary, extant research suggests that beta oscillations may provide a mechanism for coordinating auditory and motor components of the cortico-BG loop when producing speech sequences that are internally timed, and this mechanism may be impaired in stuttering speakers. Future studies investigating this aspect of stuttering should provide more fine-grained temporal information on how the basal ganglia and its connectivity with cortical areas differ in stuttering. This information can provide the basis for intervention development, which may involve better synchronizing and in turn inducing better communication across the basal ganglia, motor, and auditory regions to help achieve more fluent speech in people who stutter.

### Animal Models and Genetics Investigations Into the Neurobiological Bases of Stuttering

Speech production abilities in humans are uniquely complex, making it difficult to study its mechanisms in an animal model. However, research involving animal models – in particular those that present with learned vocal abilities – presents critical opportunities to investigate the biological mechanisms relevant to stuttering in a tractable model. As mentioned in a previous section, song nuclei and the connectivity among vocal learning pathways found in songbirds have important parallels to brain areas and neural pathways supporting human speech production (Pidoux et al., 2018; Schneider and Mooney, 2018; Jarvis, 2019). Selective manipulation or lesioning of specific regions within the songbird basal ganglia-thalamocortical homologue pathway has been shown to induce stuttering in songbirds (Kubikova et al., 2014; Chakraborty et al., 2017). The specific regions affected that led to stuttering included premotor cortex and basal ganglia (striatum) homologues, which coincide with findings in human stuttering literature that have reported neuroanatomical differences in these regions in people who stutter (Giraud et al., 2008; Chang and Zhu, 2013; Garnett et al., 2018). Thus, hypotheses guided by the DIVA model that tests aberrant function in specific nodes along the cortico-BG circuits as reviewed in previous sections of this paper may be feasible by manipulating analogous songbird neural pathways and examining behavioral changes in song structure that may parallel stuttering in humans.

Apart from songbirds, mouse vocalizations have been studied as a possible animal model for human speech as well. Mouse vocalizations are innate rather than learned (Mahrt et al., 2013) and differ from humans and songbirds in that their ventral (laryngeal) motor cortex homologue is not necessary for producing vocalizations (though necessary for pitch modulation) and the region is embedded in a non-vocal motor area (Jarvis, 2019). However, mice vocalizations can comprise complex strings of variable syllables and are used in various social situations (Schneider and Mooney, 2018). In a recent study, Barnes et al. (2016) examined vocalization changes of mice with a knock-in mutation of a stuttering related gene, GNTAB. Compared to mice without the gene mutation, mice with the GNTAB mutation exhibited increased instances of long pausing and fewer vocalizations but no differences in non-vocal behaviors. In another study from the same group, Han et al. (2019) showed that mice with GNTAB mutations and resultant vocal deficits exhibited a specific abnormality in astrocytes, and the astrocyte pathology was primarily found in the corpus callosum. The differences in vocal characteristics, brain anatomy, and structure of vocal organs limit comparisons with human stuttering, but these studies provide initial support for using mice as an animal model for stuttering. A strength to considering mice as animal models is the substantial genetic and neurobiological tools that are available, which may translate into helping advance our understanding of cellular and molecular changes associated with stuttering.

The genes identified so far associated with persistent developmental stuttering include not only GNTAB mentioned above but also GNTPG, NAGPA, and AP4E1 (Kang et al., 2010; Raza et al., 2015), which have been reported to cumulatively account for 12–20% of all stuttering cases (Frigerio-Domingues and Drayna, 2017). This group of genes plays a role in lysosomal enzyme trafficking, a basic cellular housekeeping function. How mutations in these genes specifically affect stuttering is unclear. A couple of recent papers that examined the expression patterns of these genes across brain areas, in conjunction with brain morphometric (Chow et al., 2019) and functional network differences in stuttering (Benito-Aragón et al., 2019), provide novel insights into how these genes might affect brain anatomy and function in speakers who stutter. Specifically, spatial correspondence between areas of high stuttering gene expression and anomalous brain anatomy/function converged in perisylvian areas including the left motor and auditory cortex. Group differences in gray matter volume also showed high spatial correlation with expression patterns of the GNTPG (Chow et al., 2019; subcortical areas were not included in the analysis for Benito-Aragón et al., 2019). There are limited data available to date that provide definitive links between the neurobiology of the stuttering genes and the proposed basal ganglia circuitry and mechanisms discussed in this manuscript. We expect that it is likely that more genes will be discovered in association with stuttering. Understanding the function of these genes in relation to neural circuit development relevant to stuttering will lead to more insights into the pathomechanisms underlying stuttering the future.

### Limitations of a Basal-Ganglia-Centric View of Stuttering

Although the studies reviewed herein provide broad support for basal ganglia-thalamocortical loop involvement in PDS, it is noteworthy that differences between fluent and stuttering individuals have been found for neural structures not in this loop, most notably the cerebellum (e.g., Connally et al., 2014; Yang et al., 2016), including numerous studies suggesting cerebellum-related mechanisms in compensation for stuttering (Lu et al., 2009; Etchell et al., 2014; Toyomura et al., 2015; Sitek et al., 2016; Kell et al., 2018). Importantly, the cerebellum and basal ganglia are interconnected at the subcortical level, with cerebellar output nuclei projecting to the striatum through a dense disynaptic projection (Bostan and Strick, 2018), suggesting the possibility that cerebellar projections to basal ganglia and/or cerebral cortex may provide a means for compensating for impaired basal ganglia function in stuttering. More generally, although the model described herein provides a comprehensive account of a wide range of data concerning the neural bases of stuttering, much work remains to be done to verify many details of this account, as well as to account for how brain regions not treated by our model (such as the cerebellum) impact stuttering behaviors.

## Conclusions

The basal ganglia and their connections to cortical regions involved in speech form critical networks that support fluent speech production. Anatomy and function of this cortico-BG loop have been found to be atypical in an increasing number of studies of speakers who stutter, pointing to possible deficits within the basal ganglia proper; connections between cortex, basal ganglia, and thalamus; and in the cortical circuitry involved in speech production. Future studies that examine in greater detail neurological deficits in the morphology, interconnectivity, functionality, and developmental time course of the cortico-BG network have great potential to further our knowledge on possible neural vulnerabilities for chronic stuttering and for distinguishing core deficits from anomalies/compensatory deficits that develop after years of stuttering. These studies will in turn help pave the way to developing neuroscience-guided treatments for stuttering that may not only help alleviate stuttering in adults who stutter but also help prevent chronic stuttering during childhood with early intervention.

## Author Contributions

S-EC and FG planned, drafted, and wrote the manuscript.

### Conflict of Interest

The authors declare that the research was conducted in the absence of any commercial or financial relationships that could be construed as a potential conflict of interest.

## Footnotes

^1^Impaired initiation and/or termination of movements in PDS has also been reported in a number of non-speech tasks such as auditory tracking (Nudelman et al., 1992) or producing a minimal displacement of the fingers or speech articulators (De Nil and Abbs, 1991; Howell et al., 1995; Loucks and De Nil, 2006).

^2^The small sample of PWS in this study, coupled with the lack of published follow-up studies, suggests caution in interpreting these experimental findings. In particular, excess striatal dopamine might be representative of only one subtype of stuttering, as discussed later in this section.

^3^Unfortunately, these drugs typically have serious side effects that often outweigh improvements in fluency in most study participants, as detailed in Bothe et al. (2006).

^4^To measure gyrification, we used the FreeSurfer (Fischl, 2012) tool to extract the local gyrification index (lGI), which quantifies the amount of cortex within sulcal folds relative to that of the outer cortex.

## Abbreviations

AF: Arcuate fasciculus
BA: Brodmann area
CST: Corticospinal tract
CWS: Children who stutter
dIFo: Inferior frontal gyrus pars opercularis, dorsal division
dMC: (Primary) motor cortex, dorsal division
DWI: Diffusion weighted imaging
FA: Fractional anisotropy
FDR: False discovery rate
FDT: FMRIB’s diffusion toolbox
FWE: Family-wise error
GM: Gray matter
IFo: Inferior frontal gyrus, pars opercularis
MNI: Montreal Neurological Institute
NBS: Network-based statistic
PDS: Persistent developmental stuttering
PFS: Person(s) with fluent speech
PWS: Person(s) who stutter
ROI: Region of interest
SLP: Speech language pathologist
SSI: Stuttering severity instrument
TBSS: Tract-based spatial statistic
TE: Echo time
TFCE: Threshold-free cluster enhancement
TI: Inversion time
TR: Repetition time
WM: White matter.
